# 58. Impact of Order-set Modifications and Provider Education on Broad-Spectrum Antibiotic Use in Patients Admitted with Community Acquired Pneumonia

**DOI:** 10.1093/ofid/ofab466.260

**Published:** 2021-12-04

**Authors:** Jessica L Colmerauer, Kristin Linder, Casey J Dempsey, Joseph L Kuti, David P Nicolau, Anastasia Bilinskaya

**Affiliations:** 1 Virtua Health, Cherry Hill, New Jersey; 2 Hartford HealthCare, Hartford, Connecticut; 3 Center for Anti-Infective Research and Development, Hartford Hospital, Hartford, Connecticut; 4 Hartford Hospital, Hartford, Connecticut

## Abstract

**Background:**

Following updates to IDSA guidelines in 2019, Hartford HealthCare implemented changes to the community acquired pneumonia (CAP) order-set in August 2020 to reflect criteria for prescribing of broad-spectrum antimicrobial therapy. The objective of the study was to evaluate changes in broad-spectrum antibiotic days of therapy (DOT) following these order-set updates with accompanying provider education.

**Methods:**

This was a multi-center, quasi-experimental, retrospective study of patients with CAP from 9/1/19 to 10/31/19 (pre-intervention) and 9/1/20 to 10/31/20 (post-intervention). Patients were identified using ICD-10 codes indicating lower respiratory tract infection and excluded if had a positive SARS-COV-2 PCR during admission. Data collected included demographics, labs and vitals, radiographic, microbiological, and antibiotic data. The primary outcome was change in broad-spectrum antibiotic DOT, specifically anti-pseudomonal β-lactams and anti-MRSA antibiotics. Secondary outcomes included guideline-concordance of initial antibiotics, utilization of an order-set to prescribe antibiotics, and length of stay (LOS).

**Results:**

A total of 331 and 352 patients were included in the pre- and post-intervention groups, respectively. The overall duration of broad-spectrum therapy was a median of 2 days (IQR 0-8 days) in the pre-intervention period and 0 days (IQR 0-4 days) in the post-intervention period (p< 0.001). Patients in whom the order-set was used in the post-intervention period were more likely to have guideline-concordant regimens ([36/40] 90% vs. [190/312] 60.9%; p = 0.003). There were no differences in order set usage (10% vs. 11.3%, p = 0.642) between the pre- and post-intervention groups, respectively. Hospital LOS was lower in the post-intervention cohort (4.8 days [2.9-7.2 days] vs. 5.3 days [IQR 3.5-8.5 days], p = .002).

**Conclusion:**

Despite low utilization of the order-set, education surrounding order-set changes appeared to improve antibiotic prescribing and hospital LOS in our population. Further opportunities to improve order-set use and thus further increase guideline-concordant therapy are still available.

**Disclosures:**

**Joseph L. Kuti, PharmD**, **Allergan** (Speaker’s Bureau)**BioMérieux** (Consultant, Research Grant or Support, Speaker’s Bureau)**Contrafect** (Scientific Research Study Investigator)**GSK** (Consultant)**Merck** (Research Grant or Support)**Paratek** (Speaker’s Bureau)**Roche Diagnostics** (Research Grant or Support)**Shionogi** (Research Grant or Support)**Summit** (Scientific Research Study Investigator) **David P. Nicolau, PharmD**, **Abbvie, Cepheid, Merck, Paratek, Pfizer, Wockhardt, Shionogi, Tetraphase** (Other Financial or Material Support, I have been a consultant, speakers bureau member, or have received research funding from the above listed companies.)

